# Effect of Age on Glasgow Coma Scale in Patients with Moderate and Severe Traumatic Brain Injury: An Approach with Propensity Score-Matched Population

**DOI:** 10.3390/ijerph14111378

**Published:** 2017-11-13

**Authors:** Cheng-Shyuan Rau, Shao-Chun Wu, Yi-Chun Chen, Peng-Chen Chien, Hsiao-Yun Hsieh, Pao-Jen Kuo, Ching-Hua Hsieh

**Affiliations:** 1 Department of Neurosurgery, Kaohsiung Chang Gung Memorial Hospital, Chang Gung University College of Medicine, Kaohsiung 83301, Taiwan; ersh2127@cloud.cgmh.org.tw; 2 Department of Anesthesiology, Kaohsiung Chang Gung Memorial Hospital, Chang Gung University College of Medicine, Kaohsiung 83301, Taiwan; shaochunwu@gmail.com; 3 Department of Plastic Surgery, Kaohsiung Chang Gung Memorial Hospital, Chang Gung University College of Medicine, Kaohsiung 83301, Taiwan; libe320@yahoo.com.tw (Y.-C.C.); VENU_CHIEN@hotmail.com (P.-C.C.); sylvia19870714@hotmail.com (H.-Y.H.); bow110470@gmail.com (P.-J.K.)

**Keywords:** traumatic brain injury (TBI), Glasgow Coma Scale (GCS), age, elderly, propensity-score matching

## Abstract

*Background:* The most widely used methods of describing traumatic brain injury (TBI) are the Glasgow Coma Scale (GCS) and the Abbreviated Injury Scale (AIS). Recent evidence suggests that presenting GCS in older patients may be higher than that in younger patients for an equivalent anatomical severity of TBI. This study aimed to assess these observations with a propensity-score matching approach using the data from Trauma Registry System in a Level I trauma center. *Methods:* We included all adult patients (aged ≥20 years old) with moderate to severe TBI from 1 January 2009 to 31 December 2016. Patients were categorized into elderly (aged ≥65 years) and young adults (aged 20–64 years). The severity of TBI was defined by an AIS score in the head (AIS 3‒4 and 5 indicate moderate and severe TBI, respectively). We examined the differences in the GCS scores by age at each head AIS score. Unpaired Student’s *t*- and Mann–Whitney *U*-tests were used to analyze normally and non-normally distributed continuous data, respectively. Categorical data were compared using either the Pearson chi-square or two-sided Fisher’s exact tests. Matched patient populations were allocated in a 1:1 ratio according to the propensity scores calculated using NCSS software with the following covariates: sex, pre-existing chronic obstructive pulmonary disease, systolic blood pressure, hemoglobin, sodium, glucose, and alcohol level. Logistic regression was used to evaluate the effects of age on the GCS score in each head AIS stratum. *Results:* The study population included 2081 adult patients with moderate to severe TBI. These patients were categorized into elderly (*n* = 847) and young adults (*n* = 1234): each was exclusively further divided into three groups of patients with head AIS of 3, 4, or 5. In the 162 well-balanced pairs of TBI patients with head AIS of 3, the elderly demonstrated a significantly higher GCS score than the young adults (14.1 ± 2.2 vs. 13.1 ± 3.3, respectively; *p* = 0.002). In the 362 well-balanced pairs of TBI patients with head AIS of 4, the elderly showed a significantly higher GCS score than the young adults (13.1 ± 3.3 vs. 12.2 ± 3.8, respectively; *p* = 0.002). In the 89 well-balance pairs of TBI patients with head AIS of 5, no significant differences were observed for the GCS scores. *Conclusions:* This study demonstrated that elderly patients with moderate TBI present higher GCS score than younger patients. This study underscores the importance of determining of TBI severity in this group of elderly patients based on the GCS score alone. A lower threshold of GCS cutoff should be adopted in the management of the elderly patients with TBI.

## 1. Background

To date, traumatic brain injury (TBI) remains the leading cause of death and disability worldwide as well as the most important single injury contributing to traumatic mortality and morbidity [1]. Since 1974, the Glasgow Coma Scale (GCS) has been used as a triage tool to assess the severity of neurologic deficits and predict the prognosis in patients with TBI [2,3,4,5,6]. The GCS focuses on the important functions of the central nervous system, consisting of eye-opening, verbal, and motor responses, and accordingly categorizes the patients into severe (GCS score, 3–8), moderate (GCS score, 9–12), or mild TBI (GCS score, 13–15) groups [7]. Some studies reported that in addition to age and pupillary reaction, best motor response can also predict mortality [8]. Even the motor response component of the GCS alone can predict the mortality outcome in patients with TBI with nearly the same accuracy as that of the total GCS score [9,10]. GCS is used in most fields to identify patients who should likely be transferred to a neuroscience center or who require neurosurgical intervention [11]. Therefore, knowledge regarding the correlations between GCS scores and potential patient outcomes would aid physicians and care providers in dealing with the patients with TBI.

However, one disadvantage of the GCS is that the summed score does not always depict the patient’s condition accurately [12,13]. Furthermore, recent studies suggest that for an equivalent anatomical severity of TBI, the elderly may present with a higher GCS than the younger patients [4,8,14,15]. Compared to younger patients, the distribution of presenting GCS was significantly higher in the overall elderly patients, and also at each given anatomical severity [14]. Elderly patients may have severe anatomical TBI with high ensuing mortality despite the presentation with a near-normal GCS [16]. In elderly patients with trauma, decreases of GCS scores from 15–14 and 14–13 were associated with a significant 1.4-fold (95% confidence interval (CI) = 1.07–1.83) and 2.3-fold (95% CI = 1.57–3.52), respectively, odds of mortality than those of the younger patients [17]. Although elderly patients had been reported to have the most severe TBI, accompanied by an in-hospital mortality of 49%, the median presenting GCS was 14 [15].

Therefore, age may affect the relationship between anatomic severity of TBI and the neurologic conditions measured by the GCS, even after adjusting the covariates of vital signs and demographic characteristics [4]. However, these studies did not compare the patients by adjusting some other potential confounding factors that may also impact the evaluation of GCS, i.e., low hemoglobin (Hb) after a hemorrhage [18,19], profound hyponatremia [20,21,22], hypoglycemia [23,24], and alcohol intoxication [25,26]. In addition, whether the motor response of GCS is higher in the elderly when compared to the younger patients for a given anatomical severity of TBI remains unclear. Therefore, the present study aimed to assess the effects of age on the scoring of GCS and its motor response components in patients with TBI. Here, we focused on patients with moderate to severe TBI and selected a propensity score-matched patient cohort to reduce the effects of differences in sex, systolic blood pressure (SBP), Hb, sodium (Na), glucose, and blood alcohol concentration (BAC) in the patient population on the outcome assessment. We hypothesized that neurologic deficit, defined by the GCS or its motor response components, differs for elderly patients with TBI compared with younger patients with a similar anatomic TBI severity.

## 2. Methods

### 2.1. Study Population

**Figure 1:**
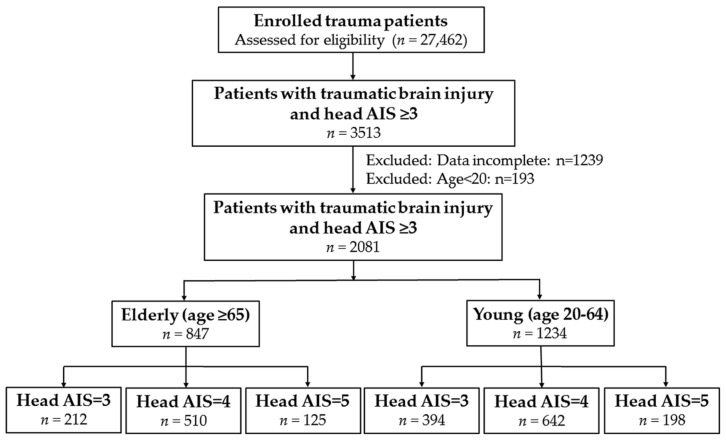
Flowchart on the allocation of patients with moderate and severe traumatic brain injuries into six exclusive groups according to age and head AIS scores.

This study was approved by the Institutional Review Board (IRB) of Kaohsiung Chang Gung Memorial Hospital, a Level I regional trauma center in southern Taiwan with reference number 201701323B0. According to IRB regulations, the requirement for informed consent was waived. We included all adult patients (≥20 years old) with moderate to severe TBI from 1 January 2009, to 31 December 2016, and were entered into the Trauma Registry System of the hospital [27,28]. The following TBIs were defined provided the following diagnostic injury codes from the *International Classification of Diseases, 9th Revision, Clinical Modification (ICD-9-CM)* were found: concussion (850.0–850.99); cerebral or cerebellar contusion or laceration (851.0–851.99); subarachnoid hemorrhage (852.0–852.19); subdural hemorrhage (852.2–852.39); extradural hemorrhage (852.4–852.59); other unspecified intracranial hemorrhage (853.0–853.19); and intracranial injury of other and unspecified nature (854.0–854.19). The Abbreviated Injury Scale (AIS) is an internationally accepted anatomy-based measurement of injury severity with a simple numeric method for ranking specific injuries in an individual [29]. The AIS assess the severity of the anatomical injury representing with minor injury (1), moderate injury (2), serious to critical (3–5), and maximal injury (6), which indicates the survival status of the patient. In this study, the severity of TBI was defined using an Abbreviated Injury Scale (AIS) score in the head (AIS 3‒4 and 5 indicate moderate and severe TBI, respectively) [30]. Patients with incomplete data were excluded. Patients were categorized into elderly (≥65 years) and young adults (20–64 years) each with grades (3–5) of AIS head injury. Finally, the study population included 2081 adult patients with moderate to severe TB: elderly (*n* = 847) and young adults (*n* = 1234), each was exclusively divided into three groups: head AIS of 3 (elderly, *n* = 212; young, *n* = 394), head AIS of 4 (elderly, *n* = 510; young, *n* = 642), and head AIS of 5 (elderly, *n* = 125; young, *n* = 198) (Figure 1). The GCS scores between these groups were compared based on each grade of AIS head injury severity. In the present study, the enrolled patients were divided into four exclusive groups based on the above criteria. The retrieved patient information for this study included the following: age; sex; comorbidities such as diabetes mellitus (DM), hypertension (HTN), coronary artery disease (CAD), congestive heart failure (CHF), cerebral vascular accident (CVA), end-stage renal disease (ESRD), and chronic obstructive pulmonary disease (COPD); injury mechanisms; SBP, Hb, Na, glucose, and BAC measured upon arrival at the emergency department; GCS scale and its motor response scores; ISS, which was expressed as the median and interquartile range (IQR, Q1–Q3); and in-hospital mortality. A BAC level of 50 mg/dL, which is the legal limit for drivers in Taiwan, was defined as the cutoff value of alcohol intoxication.

### 2.2. Statistical Analysis

The statistical analyses were performed using IBM SPSS Statistics for Windows, version 20.0 (IBM Corp., Armonk, NY, USA) and NCSS 10 software (NCSS Statistical Software, Kaysville, UT, USA). We examined the differences in GCS scores by age at each head AIS score, and this score was considered as the primary end-point. The motor response scores of GCS were also evaluated. Two-sided Fisher’s exact or Pearson chi-square tests were used to compare categorical data, and presented with the odds ratios (ORs) with 95% confidential intervals (CIs) of the calculations. The normally and non-normally distributed continuous distributed data were analyzed using unpaired Student’s *t*-tests and Mann–Whitney *U*-tests, respectively. All continuous data were presented as mean ± standard deviation. To minimize the potential confounding effects of the compared patient populations due to a non-randomized assignment, a 1:1 propensity score-matched study group (elderly vs. young adults) was created using the Greedy method with a 0.2 caliper width using NCSS 10 software. The propensity scores were calculated using a logistic regression model with the following covariates: sex, pre-existed COPD, SBP, Hb, Na, glucose level, and alcohol level. After adjusting for these confounding factors, the binary logistic regression analysis was used to evaluate the effects of age in each head AIS stratum on the GCS scores. Statistical significance was set at *p*-values of <0.05 for each analysis.

## 3. Results

### 3.1. Characteristics and Outcomes of TBI Patients with Head AIS of 3

Table 1 shows that no significant differences in sex was observed between the elderly and young adult TBI patients with head AIS of 3. DM, HTN, CAD, CHF, CVA, and COPD were significantly higher in elderly than in the young adults, but there was no difference in regard to the ESRD rates between the two groups. Compared to the young adults, many elderlies were more likely to be injured in a fall, bicycle accidents, and as a pedestrian, but only a few sustained injuries as a driver in a motorcycle accident. The elderly presented with higher SBP, lower Hb and Na levels, and lower alcohol intoxication rates than those with the young adults. The glucose level and the mean alcohol level of alcohol-intoxicated patients were not significantly different between the elderly and young adults. The elderly had a significantly higher GCS score than the young adults had (14.1 ± 2.0 vs. 12.9 ± 3.4, respectively; *p* < 0.001), with a >1 score difference. Fewer elderly patients had a GCS of ≤8, while more had a GCS score ≥ 13 than the younger patients. In addition, the motor response scores were higher in the elderly than young adult patients (5.7 ± 0.7 vs. 5.4 ± 1.1, respectively; *p* < 0.001). Elderly patients had a significantly lower ISS (median (QR: Q1–Q3), 9 [9,10,11,12,13]) than that of the young adults (13 [9,10,11,12,13,14]). However, differences regarding the mortality were not observed between the elderly and young adult patients (3.3% vs. 2.0%, respectively; *p* = 0.412).

### 3.2. Characteristics and Outcomes of TBI Patients with Head AIS of 4

Table 2 shows that the female sex was significantly predominant among the elderly than in the young adult TBI patients with head AIS of 4. Rates of DM, HTN, CAD, CVA, ESRD, and COPD were significantly higher in elderly than in young adult patients; however, there was no difference of the rate of CHF between the elderly and the young adult patients. Compared to the young adult patients, several elderly patients were injured in a fall or bicycle accidents, but fewer elderly sustained injuries as a passenger of motor vehicle, as a driver in the motorcycle accident, and by a strike by/against injury. Elderly patients presented with higher SBP, lower Hb and Na levels, and lower alcohol intoxication rates than their younger counterparts. The glucose and the mean alcohol levels of alcohol-intoxicated patients were not significantly different between the two groups. Elderly patients had a significantly higher GCS scores than that of the young adult patients (13.1 ± 3.2 vs. 11.8 ± 3.9, respectively; *p* < 0.001), with the score difference of >1. Fewer elderly patients had a GCS of ≤8 than their younger counterparts. In addition, the score for motor response was higher in elderly than that in young adult patients (5.4 ± 1.2 vs. 5.1 ± 1.3, respectively; *p* < 0.001). Elderly patients had a significantly lower ISS (16 [16,17,18,19,20]) compared to the young adult patients (18 [16,17,18,19,20,21]). A 1.7-fold odds of mortality was observed in the elderly when compared to the young adult patients (OR 1.7; 95% CI: 1.03–2.80; *p* = 0.042).

### 3.3. Characteristics and Outcomes of TBI Patients with Head AIS of 5

Table 3 shows that the female sex were significantly predominant among the elderly than in the young adult TBI group with head AIS of 5. Elderly patients had significantly higher rates of DM, HTN, CAD, and CVA compared to the young adult patients; however, the rates of CHF, ESRD, and COPD were not significantly different between the two groups. Compared to the young adult patients, many elderlies were injured in a fall accident, but fewer sustained injuries as a driver in a motorcycle accident. Elderly patients presented with higher SBP, lower Hb and Na levels, and lower rates of alcohol intoxication than their younger counterparts. The glucose and the mean alcohol levels of alcohol-intoxicated patients were not significantly different between the two groups. The GCS scores between the elderly and young adult patients (7.1 ± 4.3 vs. 6.4 ± 3.8, respectively; *p* = 0.158) were not significantly different, regardless of the GCS score stratum. No significant differences were observed in the motor response scores between the elderly and the young (3.2 ± 1.9 vs. 3.0 ± 1.8, respectively; *p* = 0.435). Elderly patients had a significantly lower ISS (25 [25,26,27,28,29]) than young adult patients had (25 [25,26,27,28,29,30,31,32,33]). A 1.8-fold odds of mortality was observed in the elderly when compared to the young adult patients (OR 1.8; 95% CI: 1.15–2.86; *p* = 0.012).

### 3.4. Comparison of Propensity-Score Matched Patients with Different Head AIS

Propensity score-matched patients were selected to reduce the effects of differences in sex, pre-existed COPD, SBP, Hb, Na, glucose, and alcohol levels on the assessment of GCS scores of the patient population. The covariates were insignificantly different between these two patient cohorts in the selected 162, 362, and 89 well-balanced pairs of TBI patients with head AIS of 3 (Table 4), 4 (Table 5), and 5 (Table 6). The logistic regression analysis of these pairs of patients showed that in the TBI patients with head AIS of 3, elderly patients had a significantly higher GCS score compared to their younger counterparts (14.1 ± 2.2 vs. 13.1 ± 3.3, respectively; *p* = 0.002), with the score difference of 1. In addition, fewer elderly had a GCS of ≤8 than that of the young adult patients. The motor response scores are higher in the elderly than that in young adult patients (5.7 ± 0.8 vs. 5.5 ± 1.0, respectively; *p* = 0.029). In the TBI patients with head AIS of 4, elderly patients had a significantly higher GCS score than that of the young adult patients (13.1 ± 3.3 vs. 12.2 ± 3.8, respectively; *p* = 0.002), with the score difference of <1. In addition, fewer elderly patients had a GCS of ≤8 than the young patients. The motor response scores are not significantly higher in the elderly patients than that in young adult patients (5.4 ± 1.2 vs. 5.2 ± 1.3, respectively; *p* = 0.053). In the TBI patients with head AIS of 5, the GCS scores, percentage of patient according to GCS stratum, and motor response scores were insignificantly different between the elderly and young adult patients.

## 4. Discussion

In the present study, we demonstrate that elderly patients have higher GCS scores than younger patients with similar moderate anatomic severity of moderate TBI, which were defined by an AIS of 3 or 4. However, this difference was not observed in patients with severe anatomic severity TBI, with an AIS of 5. Even in the selected propensity score-matched patients adjusted for the differences in sex, pre-existed COPD, SBP, Hb, Na, glucose, and alcohol levels, our study demonstrates similar conclusion that, while controlling variables, being older increased the likelihood of a better GCS score for moderate anatomic severity TBI, but not for severe TBI.

Currently, the mechanisms behind the discrepancy of observed GCS scores between the elderly and young adult patients for similar anatomical severity of TBI are unknown. It had been speculated that a greater amount of intracranial hematoma and edema occurred before the intracranial pressure increases and GCS falls [14]. According to this speculation, elderly patients should truly tolerate equivalent injuries better. However, this theory is not supported by literatures demonstrating worse outcomes in elderly than younger trauma patients, despite apparently with lower injury burden [31,32,33]. In addition, such unfavorable outcome was observed with an increase in age at every decade [34]. Some studies suggested that, compared to the young patients, elderly patients with TBI are more likely to have suffered a subdural hematoma, which tend to evolve more slowly and may result in a higher initial GCS on presentation [35]. However, there is lack of evidence regarding the influence of age on GCS is exerted through preponderance on one particular type of intracranial injury in elderly patients [4]. Furthermore, some authors suggested that elderly patients with TBI are more likely to be women, and the female sex somehow confers a degree of neuroprotection in isolated TBI [14]. However, such speculation still cannot depict the relationship between neuroprotection and initial measurement of the GCS score. Similarly, our results also did not support the hypothesis that the GCS score overestimates the severity of TBI in younger patients, owing to the alcohol-related impairment. In addition, some authors proposed that the GCS score underestimates the severity of injury in elderly patients, owing to the clinician’s perception of lower baseline neurologic response [36]. Because the mechanisms behind are unknown, this study could not explain why the GCS scores and motor response components of the elderly are better than those of the younger patients with similar anatomic severity of moderate TBI, but not of severe TBI.

Our study suggests that a bias exists in the patients with moderate TBI defined using the GCS, such that elderly patients in each GCS stratum may have worse anatomic injuries than their younger counterparts. In this study, the GCS scores of the elderly TBI patients with head AIS of 3 and 4 were 14.1 ± 2.0 and 13.1 ± 3.2, respectively, which were higher by >1 score than their younger counterparts. Therefore, the elderly population may require an alternative strategy by not only solely relying on the GCS score to ensure the appropriateness of decisions made regarding the severity of illness, triage protocol, and requirement of a transfer. Moore and colleagues suggested that all head trauma patients aged ≥65 years presenting a GCS score of >13 should undergo a screening computed tomography of the head [37]. The Canadian CT Head Rule recommends that patients aged ≥65 is a high risk factor for neurosurgical intervention and recommend head computed tomography for those with GCS score of 13–15 [38]. The Ohio Emergency Medical Services system had adopted a lower threshold of GCS cutoff for elderly patients for prompt transport to a trauma center [17]. The change of the EMS trauma triage cutoff for elderly patients from GCS 13 to 14 results in improved sensitivity for clinically relevant outcomes [17]. In addition, in this study on patients with moderate to severe TBI, the motor response component of the GCS yields similar prediction rates as those of the summed GCS score, which was in accordance with the report by McNett et al. [6] and Potter et al. [16], the latter had chosen a GCS motor score of <4 as the threshold for transportation directly to the major trauma center.

This study had some limitations that should be acknowledged. First there was an inherent selection bias associated with the retrospective study design, which had the potential disadvantages include the possibility of selection bias and the effect of drugs, dyslipidemia, or therapeutic intervention on GCS scoring could not be evaluated because of insufficient data. Besides, a strong collaboration between the physician and the care manager can attribute to the outcomes of the patients [39]. In this study, the agreement in GCS scoring between different physicians may influence the outcomes of this study; however, this difference could not be evaluated precisely in this retrospective study. Second, although the intubation rate by emergency medical services was very low in southern Taiwan [40,41], we did not correct the intubation status before arrival, and the GCS score may be actually lower in patients who were intubated before the GCS score was recorded at the emergency department. Third, preinjury medications, including sedative, hypnotic, and anticoagulants expected to be more commonly used in elderly patients, were unknown and thus may result in selection bias. Furthermore, in this study, the observation that the GCS appeared to be confounded by age under the assumption that a “true” assessment on the severity of injury was conducted using the anatomic (AIS) rather than the neurologic (GCS) injury measurement. However, the hypothesis that the AIS was less confounded by age than the GCS could not be validated. At last, different matching method such as coarsened exact matching may create different matched populations for estimate than the propensity-score matching used in this study, thus may lead to a bias in the outcomes reported [42].

## 5. Conclusions

With the demonstration that the elderly with moderate TBI (head AIS of 3 or 4) present with a higher GCS score than that in younger patients, this study underscores the importance of determining the TBI severity in elderly patients using the GCS only. A lower threshold of GCS cutoff should be adopted in the management of elderly patients with moderate TBI.

## Figures and Tables

**Table 1:** Comparison of the characteristics and outcomes of the elderly and young adult patients with head AIS of 3.

Variables	Elderly *n* = 212		Young *n* = 394		Odds Ratio (95% CI)		*p*
Gender, n (%)							0.548
Male	116	(54.7)	227	(57.6)	0.9	(0.64–1.24)	
Female	96	(45.3)	167	(42.4)	1.1	(0.84–1.58)	
Co-morbidities, n (%)							
DM	58	(27.4)	35	(8.9)	3.9	(2.44–6.12)	<0.001
HTN	107	(50.5)	70	(17.8)	4.8	(3.25–6.85)	<0.001
CAD	18	(8.5)	4	(1.0)	9.0	(3.02–27.10)	<0.001
CHF	6	(2.8)	1	(0.3)	11.4	(1.37–95.72)	0.009
CVA	24	(11.3)	6	(1.5)	8.3	(3.32–20.54)	<0.001
ESRD	4	(1.9)	3	(0.8)	2.5	(0.56–11.31)	0.247
COPD	5	(2.4)	1	(0.3)	9.5	(1.10–81.79)	0.022
Mechanisms, n (%)							
Driver of MV	3	(1.4)	4	(1.0)	1.4	(0.31–6.31)	0.700
Passenger of MV	0	(0.0)	6	(1.5)	–		0.096
Driver of Motorcycle	75	(35.4)	283	(71.8)	0.2	(0.15–0.31)	<0.001
Passenger of Motorcycle	4	(1.9)	12	(3.0)	0.6	(0.20–1.92)	0.443
Bicycle	18	(8.5)	11	(2.8)	3.2	(1.50–6.98)	0.003
Pedestrian	16	(7.5)	11	(2.8)	2.8	(1.29–6.24)	0.008
Fall	94	(44.3)	53	(13.5)	5.1	(3.45–7.62)	<0.001
Strike by/against	2	(0.9)	14	(3.6)	0.3	(0.06–1.15)	0.064
SBP (mmHg)	165.1	±34.8	143.7	±30.5	–		<0.001
Hb (mg/dL)	12.6	±1.9	13.6	±2.4	–		<0.001
Na (mg/dL)	137.9	±4.1	138.8	±2.8	–		0.001
Glucose (mg/dL)	156.4	±67.9	147.4	±52.9	–		0.073
Alcohol > 50, n (%)	4	(1.9)	79	(20.1)	0.1	(0.03–0.21)	<0.001
Alcohol level	158.5	±51.0	187.2	±80.9	–		0.487
GCS	14.1	±2.0	12.9	±3.4	–		<0.001
≤8, n (%)	9	(4.2)	65	(16.5)	0.3	(0.10–0.43)	<0.001
9–12, n (%)	14	(6.6)	45	(11.4)	0.5	(0.25–0.88)	0.010
≥13, n (%)	189	(89.2)	284	(72.1)	–		–
Motor response of GCS	5.7	±0.7	5.4	±1.1	–		<0.001
ISS (median, IQR)	9	(9–13)	13	(9–14)	–		0.021
Mortality, n (%)	7	(3.3)	8	(2.0)	1.6	(0.59–4.61)	0.412

CAD = coronary artery disease; CHF = congestive heart failure; CI = confidence interval; CVA = cerebral vascular accident; DM = diabetes mellitus; ESRD = end-stage renal disease; GCS = Glasgow Coma Scale; Hb = hemoglobin; HTN = hypertension; IQR = interquartile range; ISS = injury severity score; MV = motor vehicle; Na = Sodium; SBP = systolic blood pressure.

**Table 2:** Comparison of the characteristics and outcomes of the elderly and young adult patients with head AIS of 4.

Variables	Elderly *n* = 510		Young *n* = 642		Odds Ratio (95% CI)		*p*
Gender, n (%)							<0.001
Male	270	(52.9)	442	(68.8)	0.5	(0.40–0.65)	
Female	240	(47.1)	200	(31.2)	2.0	(1.54–2.50)	
Co-morbidities, n (%)							
DM	152	(29.8)	75	(11.7)	3.2	(2.36–4.36)	<0.001
HTN	263	(51.6)	129	(20.1)	4.2	(3.27–5.49)	<0.001
CAD	62	(12.2)	11	(1.7)	7.9	(4.13–15.25)	<0.001
CHF	6	(1.2)	2	(0.3)	3.8	(0.77–18.96)	0.149
CVA	61	(12.0)	12	(1.9)	7.1	(3.80–13.40)	<0.001
ESRD	27	(5.3)	11	(1.7)	3.2	(1.58–6.53)	0.001
COPD	12	(2.4)	1	(0.2)	15.4	(2.02–119.19)	<0.001
Mechanisms, n (%)							
Driver of MV	1	(0.2)	7	(1.1)	0.2	(0.02–1.45)	0.084
Passenger of MV	0	(0.0)	7	(1.1)	–		0.020
Driver of Motorcycle	155	(30.4)	379	(59.0)	0.3	(0.24–0.39)	<0.001
Passenger of Motorcycle	9	(1.8)	13	(2.0)	0.9	(0.37–2.05)	0.831
Bicycle	35	(6.9)	22	(3.4)	2.1	(1.20–3.59)	0.009
Pedestrian	21	(4.1)	25	(3.9)	1.1	(0.59–1.92)	0.880
Fall	283	(55.5)	156	(24.3)	3.9	(3.02–4.99)	<0.001
Strike by/against	6	(1.2)	33	(5.1)	0.2	(0.09–0.53)	<0.001
SBP (mmHg)	166.7	±34.6	148.7	±34.5	–		<0.001
Hb (mg/dL)	12.2	±2.0	13.5	±1.9	–		<0.001
Na (mg/dL)	137.8	±4.1	138.6	±3.7	–		0.001
Glucose (mg/dL)	165.3	±63.9	161.6	±67.9	–		0.345
Alcohol > 50, n (%)	3	(0.6)	140	(21.8)	0.02	(0.01–0.07)	<0.001
Alcohol level	237.0	±128.2	194.3	±81.2	–		0.374
GCS	13.1	±3.2	11.8	±3.9	–		<0.001
≤8, n (%)	61	(12.0)	165	(25.7)	0.4	(0.27–0.52)	<0.001
9–12, n (%)	65	(12.7)	89	(13.9)	0.7	(0.52–1.05)	0.094
≥13, n (%)	384	(75.3)	388	(60.4)	–		–
Motor response of GCS	5.4	±1.2	5.1	±1.3	–		<0.001
ISS (median, IQR)	16	(16–20)	18	(16–21)	–		<0.001
Mortality, n (%)	38	(7.5)	29	(4.5)	1.7	(1.03–2.80)	0.042

CAD = coronary artery disease; CHF = congestive heart failure; CI = confidence interval; CVA = cerebral vascular accident; DM = diabetes mellitus; ESRD = end-stage renal disease; GCS = Glasgow Coma Scale; Hb = hemoglobin; HTN = hypertension; IQR = interquartile range; ISS = injury severity score; MV = motor vehicle; Na = Sodium; SBP = systolic blood pressure.

**Table 3:** Comparison of the characteristics and outcomes of the elderly and young adult patients with head AIS of 5.

Variables	Elderly *n* = 125		Young *n* = 198		Odds Ratio (95% CI)		*p*
Gender, n (%)							<0.001
Male	64	(51.2)	144	(72.7)	0.4	(0.25–0.63)	
Female	61	(48.8)	54	(27.3)	2.5	(1.59–4.07)	
Co-morbidities, n (%)							
DM	33	(26.4)	17	(8.6)	3.8	(2.02–7.22)	<0.001
HTN	58	(46.4)	36	(18.2)	3.9	(2.35–6.45)	<0.001
CAD	18	(14.4)	3	(1.5)	10.9	(3.15–37.97)	<0.001
CHF	1	(0.8)	0	(0.0)	–		0.149
CVA	12	(9.6)	2	(1.0)	10.4	(2.29–47.33)	<0.001
ESRD	7	(5.6)	5	(2.5)	2.3	(0.71–7.38)	0.226
COPD	3	(2.4)	0	(0.0)	–		0.057
Mechanisms, n (%)							
Driver of MV	1	(0.8)	4	(2.0)	0.4	(0.04–3.54)	0.652
Passenger of MV	0	(0.0)	3	(1.5)	–		0.286
Driver of Motorcycle	35	(28.0)	111	(56.1)	0.3	(0.19–0.49)	<0.001
Passenger of Motorcycle	2	(1.6)	6	(3.0)	0.5	(0.10–2.62)	0.492
Bicycle	13	(10.4)	13	(6.6)	1.7	(0.74–3.69)	0.293
Pedestrian	5	(4.0)	11	(5.6)	0.7	(0.24–2.09)	0.608
Fall	67	(53.6)	44	(22.2)	4.0	(2.49–6.57)	<0.001
Strike by/against	2	(1.6)	6	(3.0)	0.5	(0.10–2.62)	0.492
SBP (mmHg)	176.3	±49.8	149.2	±47.5	–		<0.001
Hb (mg/dL)	11.9	±2.1	13.0	±2.2	–		<0.001
Na (mg/dL)	137.2	±5.0	138.9	±4.4	–		0.002
Glucose (mg/dL)	205.8	±97.8	200.0	±97.9	–		0.604
Alcohol > 50, n (%)	4	(3.2)	46	(23.2)	0.1	(0.04–0.31)	<0.001
Alcohol level	154.8	±63.9	200.2	±64.9	–		0.185
GCS	7.1	±4.3	6.4	±3.8	–		0.158
≤8, n (%)	85	(68.0)	154	(77.8)	0.7	(0.36–1.26)	0.256
9–12, n (%)	18	(14.4)	17	(8.6)	1.3	(0.55–3.10)	0.659
≥13, n (%)	22	(17.6)	27	(13.6)	–		–
Motor response of GCS	3.2	±1.9	3.0	±1.8	–		0.435
ISS (median, IQR)	25	(25–29)	25	(25–33)	–		<0.001
Mortality, n (%)	74	(59.2)	88	(44.4)	1.8	(1.15–2.86)	0.012

CAD = coronary artery disease; CHF = congestive heart failure; CI = confidence interval; CVA = cerebral vascular accident; DM = diabetes mellitus; ESRD = end-stage renal disease; GCS = Glasgow Coma Scale; Hb = hemoglobin; HTN = hypertension; IQR = interquartile range; ISS = injury severity score; MV = motor vehicle; Na = Sodium; SBP = systolic blood pressure.

**Table 4:** Comparison of GCS score and motor response components in the selected propensity–score matched cohort of elderly and young adult patients with head AIS of 3.

	Elderly *n* = 162		Young *n* = 162		Odds Ratio (95% CI)		*p*	Standardized Difference
Gender, n (%)					1.0	(0.65–1.54)	1.000	0.00%
Male	85	(52.5)	85	(52.5)	–		–	–
Female	77	(47.5)	77	(47.5)	–		–	–
COPD								
Yes	0	(0.0)	0	(0.0)	–		–	–
No	162	(100)	162	(100)	–		–	–
SBP (mmHg)	157.4	±31.9	156.7	±31.5	–		0.838	2.28%
Hb (mg/dL)	12.8	±1.8	12.9	±1.9	–		0.802	–2.79%
Na (mg/dL)	138.4	±3.8	138.4	±3.2	–		0.899	1.41%
Glucose (mg/dL)	158.5	±72.4	155.9	±62.0	–		0.727	3.88%
Alcohol > 50, n (%)	4	(2.5)	4	(2.5)	1.0	(0.25–4.07)	1.000	0.00%
GCS	14.1	±2.2	13.1	±3.3	–		0.002	–
≤8, n (%)	8	(4.9)	25	(15.4)	0.3	(0.12–0.63)	0.002	–
9–12, n (%)	10	(6.2)	13	(8.0)	0.7	(0.28–1.56)	0.389	–
≥13, n (%)	144	(88.9)	124	(76.5)	–		–	–
Motor response	5.7	±0.8	5.5	±1.0	–		0.029	–

CI = confidence interval; GCS = Glasgow Coma Scale; Hb = hemoglobin; Na = Sodium; SBP = systolic blood pressure.

**Table 5:** Comparison of GCS score and motor response components in the selected propensity–score matched cohort of elderly and young adult patients with head AIS of 4.

	Elderly *n* = 362		Young *n* = 362		Odds Ratio (95% CI)		*p*	Standardized Difference
Gender, n (%)					1.0	(0.75–1.34)	1.000	0.00%
Male	202	(55.8)	202	(55.8)	–		–	–
Female	160	(44.2)	160	(44.2)	–		–	–
COPD								
Yes	0	(0.0)	0	(0.0)	–		–	–
No	362	(100)	362	(100)	–		–	–
SBP (mmHg)	160.1	±31.5	158.8	±32.2	–		0.589	4.01%
Hb (mg/dL)	12.7	±1.8	12.8	±1.9	–		0.755	–2.32%
Na (mg/dL)	138.0	±3.7	138.0	±4.0	–		1.000	0.00%
Glucose (mg/dL)	170.2	±66.3	168.8	±65.0	–		0.766	2.22%
Alcohol > 50, n (%)	2	(0.6)	2	(0.6)	1.0	(0.14–7.14)	1.000	0.00%
GCS	13.1	±3.3	12.2	±3.8	–		0.002	–
≤8, n (%)	46	(12.7)	79	(21.8)	0.5	(0.34–0.77)	0.001	–
9–12, n (%)	47	(13.0)	47	(13.0)	0.9	(0.57–1.36)	0.575	–
≥13, n (%)	269	(74.3)	236	(65.2)	–		–	–
Motor response	5.4	±1.2	5.2	±1.3	–		0.053	–

CI = confidence interval; GCS = Glasgow Coma Scale; Hb = hemoglobin; Na = Sodium; SBP = systolic blood pressure.

**Table 6:** Comparison of GCS score and motor response components in the selected propensity–score matched cohort of elderly and young adult patients with head AIS of 5.

	Elderly *n* = 89		Young *n* = 89		Odds Ratio (95% CI)		*p*	Standardized Difference
Gender, n (%)					1.0	(0.55–1.82)	1.000	0.00%
Male	52	(58.4)	52	(58.4)	–		–	–
Female	37	(41.6)	37	(41.6)	–		–	–
COPD					–		–	–
Yes	0	(0.0)	0	(0.0)	–		–	–
No	89	(100)	89	(100)	–		–	–
SBP (mmHg)	167.3	±52.4	167.5	±45.0	–		0.985	–0.28%
Hb (mg/dL)	12.1	±2.2	12.1	±2.2	–		0.970	–0.57%
Na (mg/dL)	138.2	±3.9	138.1	±4.5	–		0.789	4.02%
Glucose (mg/dL)	210.0	±108.2	199.1	±89.9	–		0.470	10.86%
Alcohol > 50, n (%)	4	(4.5)	4	(4.5)	1.0	(0.24–4.13)	1.000	0.00%
GCS	7.0	±4.2	6.6	±4.0	–		0.525	–
≤8, n (%)	61	(68.5)	67	(75.3)	0.9	(0.38–1.90)	0.837	–
9–12, n (%)	13	(14.6)	8	(9.0)	1.5	(0.48–4.76)	0.569	–
≥13, n (%)	15	(16.9)	14	(15.7)	–		–	–
Motor response	3.2	±1.9	3.1	±1.8	–		0.873	–

CI = confidence interval; GCS = Glasgow Coma Scale; Hb = hemoglobin; Na = Sodium; SBP = systolic blood pressure.

## References

[1] Fu T.S., Jing R., McFaull S.R., Cusimano M.D. (2015). Recent trends in hospitalization and in-hospital mortality associated with traumatic brain injury in Canada: A nationwide, population-based study. J. Trauma Acute Care Surg..

[2] Teasdale G., Jennett B. (1974). Assessment of coma and impaired consciousness. A practical scale. Lancet.

[3] Singh B., Murad M.H., Prokop L.J., Erwin P.J., Wang Z., Mommer S.K., Mascarenhas S.S., Parsaik A.K. (2013). Meta-analysis of Glasgow coma scale and simplified motor score in predicting traumatic brain injury outcomes. Brain Inj..

[4] Salottolo K., Levy A.S., Slone D.S., Mains C.W., Bar-Or D. (2014). The effect of age on Glasgow Coma Scale score in patients with traumatic brain injury. JAMA Surg..

[5] Moore L., Lavoie A., Camden S., Le Sage N., Sampalis J.S., Bergeron E., Abdous B. (2006). Statistical validation of the Glasgow Coma Score. J. Trauma.

[6] McNett M. (2007). A review of the predictive ability of Glasgow Coma Scale scores in head-injured patients. J. Neurosci. Nurs..

[7] Teasdale G., Murray G., Parker L., Jennett B. (1979). Adding up the Glasgow Coma Score. Acta Neurochir. Suppl..

[8] Mamelak A.N., Pitts L.H., Damron S. (1996). Predicting survival from head trauma 24 hours after injury: A practical method with therapeutic implications. J. Trauma.

[9] Ross S.E., Leipold C., Terregino C., O’Malley K.F. (1998). Efficacy of the motor component of the Glasgow Coma Scale in trauma triage. J. Trauma.

[10] Healey C., Osler T.M., Rogers F.B., Healey M.A., Glance L.G., Kilgo P.D., Shackford S.R., Meredith J.W. (2003). Improving the Glasgow Coma Scale score: Motor score alone is a better predictor. J. Trauma.

[11] Sasser S.M., Hunt R.C., Faul M., Sugerman D., Pearson W.S., Dulski T., Wald M.M., Jurkovich G.J., Newgard C.D., Lerner E.B. (2012). Guidelines for field triage of injured patients: Recommendations of the National Expert Panel on Field Triage, 2011. MMWR Recomm. Rep..

[12] Bledsoe B.E., Casey M.J., Feldman J., Johnson L., Diel S., Forred W., Gorman C. (2015). Glasgow Coma Scale Scoring is Often Inaccurate. Prehosp. Disaster Med..

[13] Feldman A., Hart K.W., Lindsell C.J., McMullan J.T. (2015). Randomized controlled trial of a scoring aid to improve Glasgow Coma Scale scoring by emergency medical services providers. Ann. Emerg. Med..

[14] Kehoe A., Rennie S., Smith J.E. (2015). Glasgow Coma Scale is unreliable for the prediction of severe head injury in elderly trauma patients. Emerg. Med. J..

[15] Kehoe A., Smith J.E., Bouamra O., Edwards A., Yates D., Lecky F. (2016). Older patients with traumatic brain injury present with a higher GCS score than younger patients for a given severity of injury. Emerg. Med. J..

[16] Potter D., Kehoe A., Smith J.E. (2013). The sensitivity of pre-hospital and in-hospital tools for the identification of major trauma patients presenting to a major trauma centre. J. R. Nav. Med. Serv..

[17] Caterino J.M., Raubenolt A., Cudnik M.T. (2011). Modification of Glasgow Coma Scale criteria for injured elders. Acad. Emerg. Med..

[18] Yee K.F., Walker A.M. (2016). The Effect of Hemoglobin Levels on Mortality in Pediatric Patients with Severe Traumatic Brain Injury. Can. Respir. J..

[19] Sekhon M.S., McLean N., Henderson W.R., Chittock D.R., Griesdale D.E. (2012). Association of hemoglobin concentration and mortality in critically ill patients with severe traumatic brain injury. Crit. Care.

[20] Wu X., Lu X., Lu X., Yu J., Sun Y., Du Z., Wu X., Mao Y., Zhou L., Wu S. (2015). Prevalence of severe hypokalaemia in patients with traumatic brain injury. Injury.

[21] Meng X., Shi B. (2016). Traumatic Brain Injury Patients with a Glasgow Coma Scale Score of </=8, Cerebral Edema, and/or a Basal Skull Fracture are More Susceptible to Developing Hyponatremia. J. Neurosurg. Anesthesiol..

[22] Lohani S., Devkota U.P. (2011). Hyponatremia in patients with traumatic brain injury: Etiology, incidence, and severity correlation. World Neurosurg..

[23] Kotera A., Iwashita S., Irie H., Taniguchi J., Kasaoka S., Kinoshita Y. (2014). An analysis of the relationship between Glasgow Coma Scale score and plasma glucose level according to the severity of hypoglycemia. J. Intensiv. Care.

[24] Brady W.J., Butler K., Fines R., Young J. (1999). Hypoglycemia in multiple trauma victims. Am. J. Emerg. Med..

[25] Osler T., Cook A., Glance L.G., Lecky F., Bouamra O., Garrett M., Buzas J.S., Hosmer D.W. (2016). The differential mortality of Glasgow Coma Score in patients with and without head injury. Injury.

[26] Ronning P., Gunstad P.O., Skaga N.O., Langmoen I.A., Stavem K., Helseth E. (2015). The impact of blood ethanol concentration on the classification of head injury severity in traumatic brain injury. Brain Inj..

[27] Hsieh C.H., Hsu S.Y., Hsieh H.Y., Chen Y.C. (2017). Differences between the sexes in motorcycle-related injuries and fatalities at a Taiwanese level I trauma center. Biomed. J..

[28] Hsieh C.H., Liu H.T., Hsu S.Y., Hsieh H.Y., Chen Y.C. (2017). Motorcycle-related hospitalizations of the elderly. Biomed. J..

[29] CoMAoA, Safety (1971). Rating the severity of tissue damage. I. The abbreviated scale. JAMA.

[30] Savitsky B., Givon A., Rozenfeld M., Radomislensky I., Peleg K. (2016). Traumatic brain injury: It is all about definition. Brain Inj..

[31] Mosenthal A.C., Lavery R.F., Addis M., Kaul S., Ross S., Marburger R., Deitch E.A., Livingston D.H. (2002). Isolated traumatic brain injury: Age is an independent predictor of mortality and early outcome. J. Trauma.

[32] Mosenthal A.C., Livingston D.H., Lavery R.F., Knudson M.M., Lee S., Morabito D., Manley G.T., Nathens A., Jurkovich G., Hoyt D.B. (2004). The effect of age on functional outcome in mild traumatic brain injury: 6-month report of a prospective multicenter trial. J. Trauma.

[33] Susman M., DiRusso S.M., Sullivan T., Risucci D., Nealon P., Cuff S., Haider A., Benzil D. (2002). Traumatic brain injury in the elderly: Increased mortality and worse functional outcome at discharge despite lower injury severity. J. Trauma.

[34] Dhandapani S., Manju D., Sharma B., Mahapatra A. (2012). Prognostic significance of age in traumatic brain injury. J. Neurosci. Rural Pract..

[35] Karnath B. (2004). Subdural hematoma. Presentation and management in older adults. Geriatrics.

[36] Sheridan P.L., Hausdorff J.M. (2007). The role of higher-level cognitive function in gait: Executive dysfunction contributes to fall risk in Alzheimer’s disease. Dement. Geriatr. Cogn. Disord..

[37] Moore M.M., Pasquale M.D., Badellino M. (2012). Impact of age and anticoagulation: Need for neurosurgical intervention in trauma patients with mild traumatic brain injury. J. Trauma Acute Care Surg..

[38] Stiell I.G., Wells G.A., Vandemheen K., Clement C., Lesiuk H., Laupacis A., McKnight R.D., Verbeek R., Brison R., Cass D. (2001). The Canadian CT Head Rule for patients with minor head injury. Lancet.

[39] Ciccone M.M., Aquilino A., Cortese F., Scicchitano P., Sassara M., Mola E., Rollo R., Caldarola P., Giorgino F., Pomo V. (2010). Feasibility and effectiveness of a disease and care management model in the primary health care system for patients with heart failure and diabetes (Project Leonardo). Vasc. Health Risk Manag..

[40] Lai W.H., Rau C.S., Hsu S.Y., Wu S.C., Kuo P.J., Hsieh H.Y., Chen Y.C., Hsieh C.H. (2016). Using the Reverse Shock Index at the Injury Scene and in the Emergency Department to Identify High-Risk Patients: A Cross-Sectional Retrospective Study. Int. J. Environ. Res. Public Health.

[41] Huang C.Y., Rau C.S., Chuang J.F., Kuo P.J., Hsu S.Y., Chen Y.C., Hsieh H.Y., Hsieh C.H. (2016). Characteristics and Outcomes of Patients Injured in Road Traffic Crashes and Transported by Emergency Medical Services. Int. J. Environ. Res. Public Health.

[42] Wells A.R., Hamar B., Bradley C., Gandy W.M., Harrison P.L., Sidney J.A., Coberley C.R., Rula E.Y., Pope J.E. (2013). Exploring robust methods for evaluating treatment and comparison groups in chronic care management programs. Popul. Health Manag..

